# Robot-Assisted Ankle Rehabilitation Using the Hybrid Assistive Limb for Children after Equinus Surgery: A Report of Two Cases

**DOI:** 10.3390/pediatric14030041

**Published:** 2022-08-10

**Authors:** Kazushi Takahashi, Hirotaka Mutsuzaki, Kenichi Yoshikawa, Satoshi Yamamoto, Kazunori Koseki, Ryoko Takeuchi, Yuki Mataki, Nobuaki Iwasaki

**Affiliations:** 1 Department of Physical Therapy, Ibaraki Prefectural University of Health Sciences Hospital, 4733 Ami, Ibaraki 300-0331, Japan; 2 Department of Orthopedic Surgery, Ibaraki Prefectural University of Health Sciences Hospital, 4733 Ami, Ibaraki 300-0331, Japan; 3 Center for Medical Sciences, Ibaraki Prefectural University of Health Sciences, 4669-2 Ami, Ibaraki 300-0394, Japan; 4 Department of Physical Therapy, Ibaraki Prefectural University of Health Sciences, 4669-2 Ami, Ibaraki 300-0394, Japan; 5 Department of Rehabilitation Medicine, University of Tsukuba Hospital, 2-1-1 Amakubo, Ibaraki 305-8576, Japan; 6 Department of Pediatrics, Ibaraki Prefectural University of Health Sciences Hospital, 4733 Ami, Ibaraki 300-0331, Japan

**Keywords:** Hybrid Assistive Limb, robot-assisted training, equinus surgery

## Abstract

After equinus corrective surgery, repetitive exercises for ankle dorsiflexion and plantar flexion are crucial during rehabilitation. The single-joint Hybrid Assistive Limb (HAL-SJ) is an advanced exoskeletal robotic device with a control system that uses bioelectrical signals to assist joint motion in real time and demonstrates joint torque assistance with the wearer’s voluntary movement. We present two cases of robot-assisted ankle rehabilitation after equinus surgery using the HAL-SJ in children. Case 1 was an 8-year-old boy, whereas case 2 was a 6-year-old boy. When they were allowed to walk without braces, training with the HAL-SJ was performed postoperatively for 20 min per session a total of eight times (2–4 sessions per week). Assessments were performed before and after HAL-SJ training. During gait analysis, case 1 had improved joint angles during the stance phase on the operated side; however, case 2 had improved joint angles during the stance and swing phases. The co-activation index values of the medial gastrocnemius and tibialis anterior muscles, which were high before training, decreased after training and approached the standard value. The HAL-SJ may provide systematic feedback regarding voluntary ankle dorsiflexion and plantar flexion and is considered to have motor learning effects.

## 1. Introduction

Equinus is the most common gait disorder caused by central nervous system (CNS) disorders of pediatric patients; furthermore, it decreases the ability to participate in activities of daily living [1,2,3]. Most pediatric equinus cases are caused by CNS disorders. CNS disorders have positive features, such as spasticity, clonus, and excessive co-contraction, and negative features, such as weakness and sensory deficits. The interaction between these positive and negative features leads to musculoskeletal pathology (equinus, muscle shortening, and degenerative arthritis) [4]. Equinus is a substantial problem for children with gait disorders that must be addressed.

Conservative treatments such as physical therapy, ankle braces, casting, and botulinum toxin A injections are the treatments of choice for equinus; however, if they are not effective, then surgery may be indicated [5,6]. Orthopedic surgery performed for pediatric equinus, such as equinus correction, is an effective treatment [7]. However, immediately after orthopedic surgery to relieve spasticity of the ankle joint, correct deformities of the lower limb, and acquire new motor skills, motor function may be temporarily impaired [8]. Deterioration of function after equinus corrective surgery is caused by weakness of the released muscle and decreased physical fitness as a result of immobility of the operated limbs [8]. Therefore, after surgery, the use of the range of motion (ROM) gained in the ankle joint is important to performing repeated dorsiflexion/plantar flexion and standing balance exercises [9]. Furthermore, functional electrical stimulation (FES) has been reported to be effective for improving gait function after pediatric orthopedic surgery [10]. FES provides electrical stimulation via skin surface or implantable electrodes to cause coordinated contraction of skeletal muscles to create goal-directed movement [10,11]. However, in children, the postoperative recovery of motor function may be difficult to achieve because of pain, fear, and lack of concentration; therefore, abnormal gait, such as drooping foot, may persist. Bolton et al. reported that after a mean follow-up time of 6.9 years for children with cerebral palsy (CP) who underwent calf-lengthening surgery, 22% had recurrence of equinus (dorsiflexion was reduced), and 36% had recurrence of calcaneus (plantar flexion was reduced) [12]. This finding illustrates the difficulty and importance of postoperative rehabilitation.

**Figure 1:**
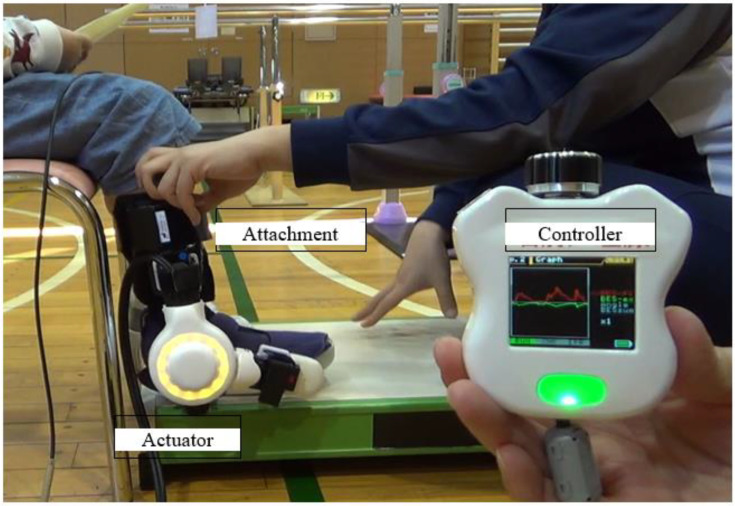
The ankle joint unit of the single-joint Hybrid Assistive Limb (HAL-FS01; Cyberdyne Inc., Tsukuba, Japan).

The single-joint Hybrid Assistive Limb (HAL-SJ) (HAL-FS01; Cyberdyne, Inc., Tsukuba, Japan) is a wearable robot that can support flexion and extension movements of various joints. The HAL-SJ has a bioelectrical signal (BES)-based control system and demonstrates joint torque assistance with the wearer’s voluntary movement. The HAL-SJ has been effectively used for knee flexion and extension exercises performed by patients who have undergone total knee arthroplasty and for elbow flexion and extension exercises performed by CP patients with spasticity [13,14]. A new ankle joint unit that makes it possible to perform ankle joint exercises has been developed as an accessory for the HAL-SJ (Figure 1). Ankle rehabilitation using the HAL-SJ has been attempted for adult patients with drop foot, and improvements in muscle activity, strength, and walking speed have been reported [15,16]. However, the results of the use of the HAL-SJ for pediatric patients who have undergone equinus surgery have not yet been reported. We believe that the HAL-SJ is an effective rehabilitation device for those who have undergone equinus corrective surgery. Therefore, we present two cases of robot-assisted ankle rehabilitation using the HAL-SJ for children after equinus surgery.

## 2. Materials and Methods

### 2.1. The Ankle HAL-SJ

The ankle HAL-SJ is a wearable exoskeletal robot that is worn on the outside of the ankle joint and uses actuators that detect BES from the tibialis anterior and gastrocnemius muscles to train the performance of plantar flexion and dorsiflexion. Additionally, light- emitting diodes are built into the joints of the robotic suit to provide visual feedback to the patient and therapist. Assist gain levels range from 0 (unassisted) to 100, thus allowing the controller to adjust the balance between flexion and extension at each level. The controller also has a monitor that displays the BES of the flexor and extensor muscles [15,16].

### 2.2. Case Presentation

#### 2.2.1. Case 1

**Figure 2:**
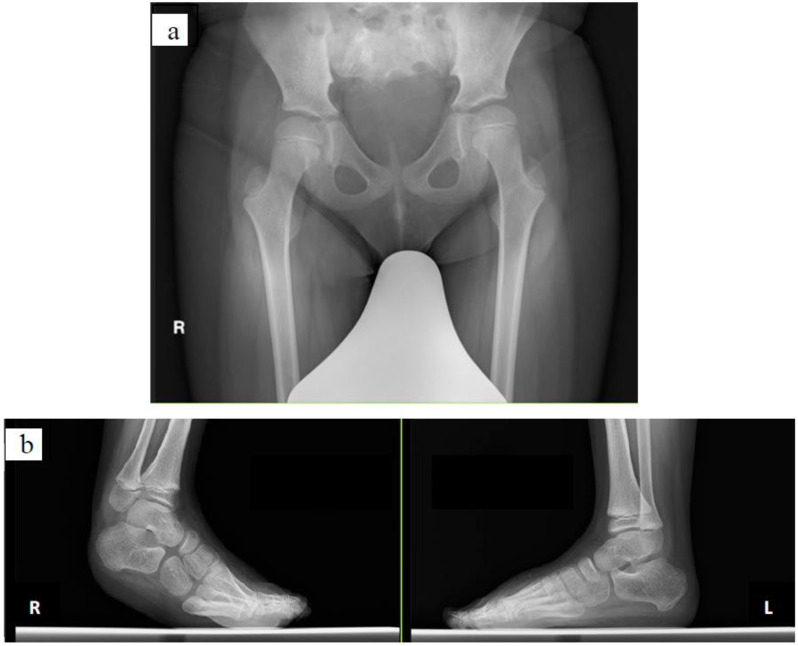
Radiographs of case 1: (**a**) pelvic radiograph and (**b**) radiograph of the feet.

An 8-year-old boy (height, 119.5 cm; weight, 29.3 kg) was diagnosed with Currarino syndrome. He had slight paraplegia with congenital partial loss of the sacrum (Figure 2a). He had more severe spasticity on the right side than on the left side, and his right equinus worsened; therefore, he underwent surgery (Figure 2b). His preoperative dorsal flexion angles were −10° on the right and 0° on the left. The patient underwent Z-lengthening of the Achilles tendon and tibialis posterior. Above-knee plaster splints were placed on his lower limbs with the knees in the extended position and ankles in the intermediate position for 2 weeks postoperatively; subsequently, below-knee plaster splints were placed for 2 weeks. Thereafter, he used short leg braces. At 40 days postoperatively, he started gait training without braces.

#### 2.2.2. Case 2

**Figure 3:**
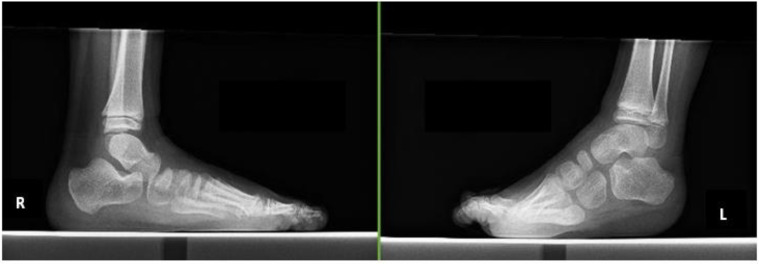
Radiographs of case 2.

A 6-year-old boy (height, 114.9 cm; weight, 23.5 kg) was diagnosed with CP, hemiplegia on the left side, and Gross Motor Function Classification System level II. He underwent surgery because of worsening left equinus (Figure 3). The preoperative dorsal flexion angles were 10° on the right and −15° on the left. He underwent selective ankle muscle release of the Achilles tendon and tibialis posterior, and the flexor digitorum longus was lengthened. His lower limbs were fixed with below-knee plaster splints for 5 weeks postoperatively; subsequently, short leg braces were used. At 50 days postoperatively, he started gait training without braces.

### 2.3. HAL-SJ Training Protocol

**Figure 4:**
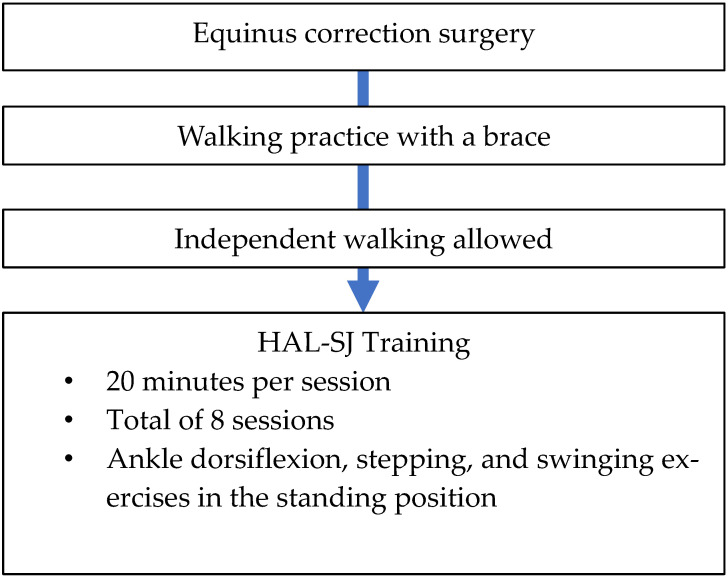
Single-joint Hybrid Assistive Limb (HAL-SJ) training protocol.

**Figure 5:**
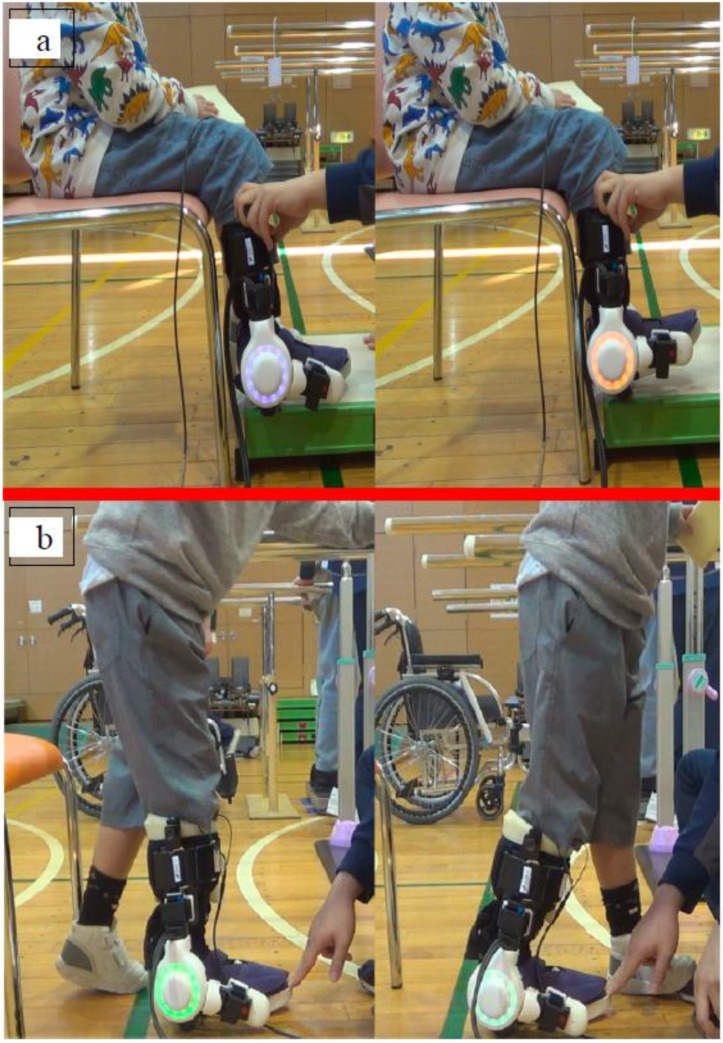
Single-joint Hybrid Assistive Limb (HAL-SJ) training: (**a**) dorsiflexion exercise and (**b**) stepping exercise.

We utilized the HAL-SJ for pediatric patients with ankle disability who underwent equinus corrective surgery. Training with the HAL-SJ was performed postoperatively when patients were allowed to walk without braces (Figure 4). We aimed to perform training with the HAL-SJ for 20 min per session for a total of eight sessions (2–4 sessions per week). Training consisted of ankle dorsiflexion, stepping, and swinging exercises in the standing position with the HAL-SJ attached to the ankle joint on the surgical side (Figure 5a,b). We performed HAL-SJ training in addition to conventional rehabilitation during hospitalization. Physical therapy and occupational therapy were performed for 40–60 min per session; five sessions were performed each week. Physical therapy included stretching, muscle strengthening exercises, physical fitness exercises, and walking practice. Occupational therapy included training to perform activities of daily living.

### 2.4. Outcome Measures

Assessments were performed before and after HAL-SJ training. End-of-training assessments were conducted within half a day to 2 days after the completion of HAL-SJ training. To determine the effectiveness of the HAL-SJ, three types of assessments were performed: clinical assessments; three-dimensional gait analysis; and analysis of the co-contraction of the medial gastrocnemius and tibialis anterior muscles.

#### 2.4.1. Clinical Assessments

We evaluated the 10 m walking test results and ROM, using the Modified Ashworth Scale (MAS) score, Selective Control Assessment of the Lower Extremity (SCALE) score, and Gross Motor Function Measure (GMFM) [17,18,19,20,21]. The 10 m walking test assessed the walking speed, length, and cadence and was performed at a selected walking speed. The ROM was measured using dorsiflexion and knee joint extension. The ROM was assessed in the supine position using goniometry, as recommended by the American Academy of Orthopaedic Surgeons [18]. The initial ROM before training was assessed by orthopedists; however, ROM before and after HAL-SJ training was assessed by a physical therapist. Spasticity of the tibialis anterior and medial gastrocnemius muscles was assessed using the MAS. Selective voluntary motor control of the lower limbs was assessed using the SCALE. Gross motor function was assessed using the GMFM, which is a rating scale used to determine the global motor function of children. The evaluation items included the following: lying down and rolling; sitting; crawling on hands and knees and kneeling; standing; and walking, running, and jumping. During this study, we mainly examined the GMFM-D score, GMFM-E score, and standing, walking, running, and jumping abilities [21].

#### 2.4.2. Three-Dimensional Gait Analysis

**Figure 6:**
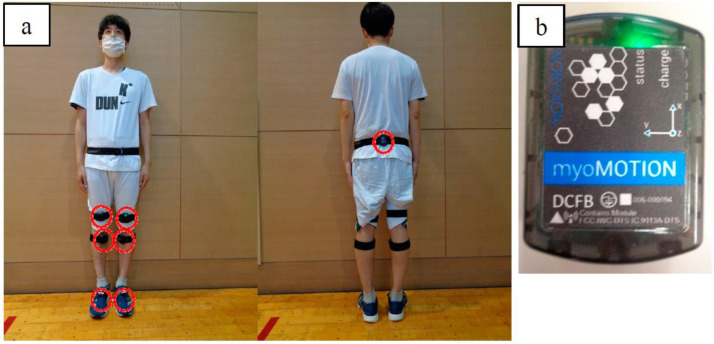
Wireless inertial measurement unit (IMU) system: (**a**) installation and (**b**) wireless IMU.

We performed a three-dimensional gait analysis to assess the joint angles of the children during the gait cycle using myoMOTION (Noraxon USA, Scottsdale, AZ, USA), a wireless inertial measurement unit (IMU) system consisting of a receiver and seven IMUs for the lower body (Figure 6a). Each IMU had a local coordinate system and measured acceleration in the following three directions: yaw, pitch, and roll (Figure 6b). Each IMU was placed on the body segments according to the lower body model provided by the IMU system software (myoRESEARCH 3.16.86, Noraxon USA, Scottsdale, AZ, USA). With this model, the body segments to which the IMUs are attached are assumed to be rigid, and each body segment is considered a rigid unit with interconnected joints. Before the measurements were performed, the system was calibrated, with children in the sitting position.

During gait analysis, 10 gait cycles obtained during the 10 m walking test were used. Gait events (initial contact and toe-off) were detected using the IMU-based contact-detection algorithm provided by myoRESEARCH. A sampling rate of 200 Hz was selected for all the IMUs. The method used by Berner et al. was used to assess the gait analysis results [22].

#### 2.4.3. Co-Contraction of the Medial Gastrocnemius and Tibialis Anterior Muscles

Co-contraction may be defined as the simultaneous activation of antagonist and agonist muscle groups in the same joint and in the same plane of movement [23]. Co-contraction is considered as a factor that contributes to recurrence of equinus after orthopedic surgery. Therefore, muscle co-contractions during ankle dorsiflexion and plantar flexion and gait were quantified using the co-activation index (COI) value determined by surface electromyography (EMG). The surface EMG activities of the medial gastrocnemius and tibialis anterior muscles were recorded during the dorsiflexion to plantar flexion and gait trials. Ankle dorsiflexion and plantar flexion test was performed 10 times on each side. COI values during walking were determined with the same method as that performed during the gait analysis. The gait cycle was categorized as follows: double stance 1 (from left heel contact to right toe-off); left stance phase (right swing phase); double stance 2 (from right heel contact to left toe-off); and right stance phase (left swing phase).

Ultium-EMG (Noraxon USA), a surface EMG, was used for EMG recordings. Surface electrodes (blue sensor; METS JPN) were used, and the distance between the electrodes was 2 cm. A sampling frequency of 2000 Hz and a bandpass filter of 20–450 Hz were used. A baseline EMG trial was conducted at rest for 1 s in the sitting position. myoRESEARCH was used to integrate the surface EMG and three-dimensional gait analysis data. 

The COI values of the tibialis anterior and medial gastrocnemius muscles were computed during the dorsiflexion to plantar flexion and gait cycles. The method used by Chow et al. was used to assess the COI values [24], which were calculated using a customized MATLAB program (MathWorks, Natick, MA, USA). Because COI values represent the relative magnitude of myoelectric overlap, it is easy to compare the resonances of different muscles [24]. The COI values range from 0 (no co-contraction) to 1 (co-contraction).

### 2.5. Ethical Considerations

This study was conducted in accordance with the Declaration of Helsinki and approved by the Ethics Committee of Ibaraki Prefectural University of Health Sciences (approval number: 797; date of approval: 28 December 2017; approval update: approval number: e356; date of approval: 8 June 2022). Informed consent was obtained in writing from the parents or guardians of the patients involved in the study, because they were minors. The research participants were also present during the explanation of the study to confirm their willingness to participate in the study.

## 3. Results

Case 2 missed one training because of family reasons. No significant deviations from the study protocol were identified, and the two participants who were trained using the HAL-SJ did not experience any serious adverse events. Table 1 summarizes the results of the clinical assessments. The results of the 10 m walking test demonstrate that both participants improved their speed and stride length.

For case 1, the walking speed increased from 0.59 to 1.05 m/s, whereas the stride length increased from 0.19 to 0.32 m. For case 2, the speed increased from 0.71 to 0.82, whereas stride length increased from 0.32 to 0.33. Moreover, for case 2, cadence improved from 131.54 to 147.06 steps/min. Furthermore, both participants improved their ROM and GMFM scores, and one participant (case 1) improved their SCALE scores. The ROM of dorsiflexion and knee joint extension on the operated side improved from 0 to 5° in case 1 and from 10 to 15 in case 2. The SCALE score improved from 10 to 12 in case 1. The GMFM score for standing improved from 79.48 to 82.05% in case 1. Regarding walking, running, and jumping, the GMFM scores improved from 54.16 to 55.55 and from 76.39 to 91.67 in case 1 and case 2, respectively.

**Figure 7:**
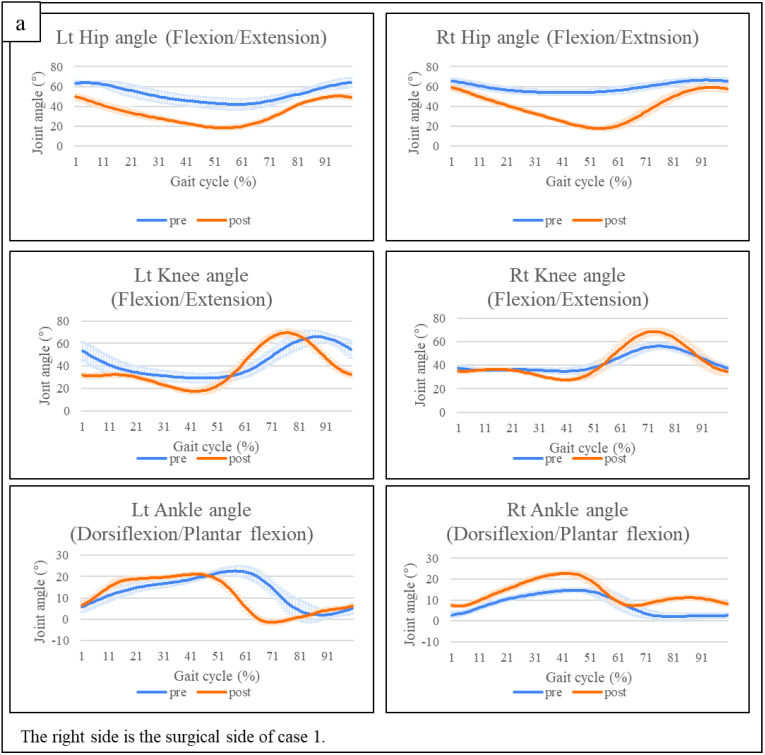

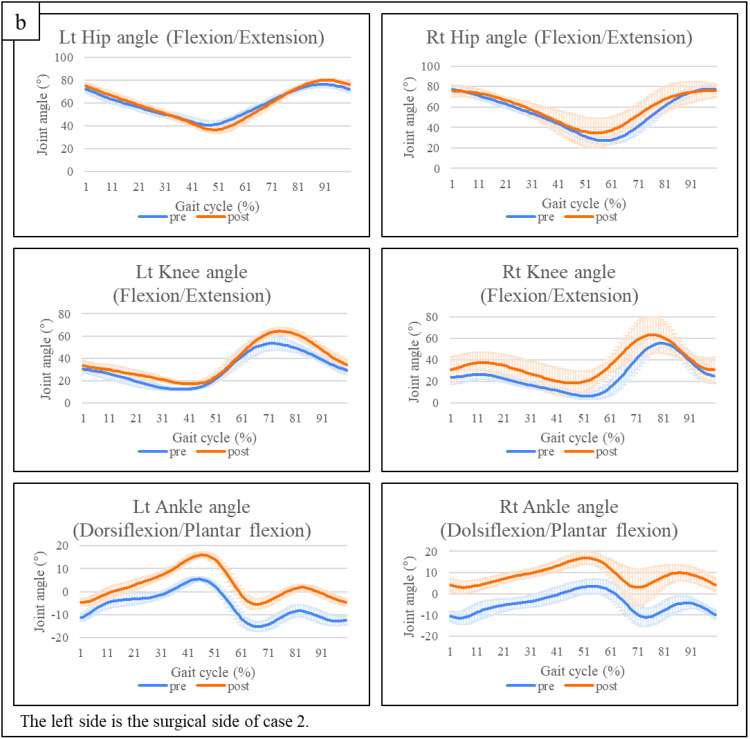
(**a**) Gait analysis of case 1. (**b**) Gait analysis of case 2. Lt: left, post: after training, pre: before training, Rt: right.

The joint angle waveforms averaged from the gait analysis results of the two participants are displayed (Table 2 and Figure 7a,b). In case 1, the joint angles of hip extension and knee extension during the stance phase on the operated side improved. The mean maximum hip extension angle ranged from −53.75 to −17.71, whereas the mean maximum knee extension angle ranged from −34.91 to −27.61. Furthermore, during the swing phase in case 1, the maximum knee flexion angle improved from 56.19 to 68.59, and the maximum ankle dorsiflexion angle improved from 4.35 to 11.20. In case 2, the joint angles of hip extension during the stance phase on the operated side improved. The mean maximum hip extension angle ranged from −41.23 to −36.57. Furthermore, during the swing phase in case 2, the maximum hip flexion angle improved from 76.43 to 80.26, and the maximum ankle dorsiflexion angle improved from −8.50 to 1.82.

The COI values of the tibialis anterior and medial gastrocnemius muscles are summarized in Table 3. In case 1, the COI value of the plantar flexion motion of the operated right ankle joint decreased from 0.98 ± 0.58 to 0.26 ± 0.12. Regarding the COI value of gait, the right side decreased from 0.76 ± 0.25 to 0.63 ± 0.24 during gait cycle 3 (double stance 2) and from 0.77 ± 0.19 to 0.48 ± 0.09 during gait cycle 4 (right standing phase). In case 2, before HAL training, the patient could not perform any dorsiflexion and plantar flexion movements of the ankle joint on the operated side, and the COI values for dorsiflexion and plantar flexion could not be calculated. After HAL training, the patient was able to perform dorsiflexion and plantar flexion exercises, and the COI value was calculated (dorsiflexion, 0.74 ± 0.27; plantar flexion, 0.34 ± 0.27). The COI value during walking slightly decreased on the operative side during the stance phase (0.68 ± 0.21 to 0.43 ± 0.06), double stance 2 (0.63 ± 0.15 to 0.55 ± 0.11), and stance phase (0.54 ± 0.12 to 0.49 ± 0.09).

## 4. Discussion

Equinus corrective surgery for children is a common and effective treatment [5,7]. However, immediately after surgery, the child’s motor function is temporarily reduced to relieve spasticity of the ankle joint [8]. Furthermore, equinus may develop again over time after surgery [12]. Therefore, postoperative rehabilitation for pediatric equinus is important. FES can effectively improve dorsiflexion and plantar flexion [10,11,25]. However, FES may be invasive [10]. Furthermore, during our clinical experience, we have found that many children who underwent electrical stimulation, including FES, are hesitant to undergo electrical stimulation because of the fear of pain. However, HAL-SJ training is a non-invasive treatment. Children who have undergone HAL-SJ training can perform repeated ankle dorsiflexion and plantar flexion exercises, which are important for improving postoperative function, without the fear associated with pain. Additionally, HAL-SJ can assist with voluntary ankle dorsiflexion and plantar flexion movements in real time using BES feedback. Furthermore, patients experience neuromuscular motor training and coordination of muscle co-contraction, which may have a motor learning effect. Therefore, the HAL-SJ could be an effective rehabilitation device after equinus corrective surgery.

Training using the HAL-SJ was conducted for two boys after equinus corrective surgery, and an improvement in motor function was achieved. The 10 m walking test is the easiest method of assessing the gait function of children [17]. During this study, walking speed and stride length improved in case 1, whereas walking speed and cadence improved in case 2. Walking speed consists of stride length and cadence. We believe that the improvements in stride length in case 1 and cadence in case 2 contributed to the improvement in walking speed. The GMFM is the gold standard for assessing gross motor skills during pediatric rehabilitation. During this study, we adopted standing, walking, running, and jumping abilities as the evaluation indices. We found that standing, walking, running, and jumping improved in case 1, and that walking, running, and jumping improved in case 2. There was no change in the standing ability in case 2. Nevertheless, the original score was 94.87%, which is close to a perfect score; therefore, we think that it had a ceiling effect. There was no change in the MAS scores before and after training with HAL-SJ. Moreover, the SCALE score of the lower limb voluntary assessment increased in case 1 and remained unchanged in case 2, indicating that HAL-SJ training did not worsen the spasticity of the lower limbs.

The results of the three-dimensional gait analysis show improved maximum hip extension and knee extension angles in the stance phase in case 1. During the equinus gait, the rocker bottom action at the heel is lost because of initial contact by the toe. This disruption of the normal sagittal-plane ankle motion contributes to the compensatory deviations at the proximal joints [26]. Goodman et al. compared gait data of healthy adult subjects with and without equinus restraints on a single ankle [27]. The results showed that ankle restraints significantly decreased during hip joint extension and knee joint extension during the stance phase on the ipsilateral side [27]. In other words, the functional improvement during this case study was attributed to improvements in ankle dorsiflexion and plantar flexion, with the positive effects extending to the proximal joint. Additionally, the typical gait cycle is characterized by a 60% stance phase (of which 20% is the double stance) and a 40% swing phase [28]. Furthermore, during a typical gait cycle, maximal knee extension in the stance phase appears during mid-stance, and maximal hip extension appears during the terminal stance phase [28]. HAL-SJ training improved the maximum angles of the hip and knee joints, similar to the joint angle curve of a typical gait cycle in case 1. The more similar to the typical gait cycle, the less energy that is required for walking [29]. The negative values improved during the swing phase of the ankle joint on the operative side in case 2. The negative value during the swing phase indicated the appearance of drop foot; however, it was improved after HAL-SJ training.

COI values can quantify co-activation using surface EMG values and range from 0 (no co-activation) to 1 (co-activation present) [24]. The COI values of the tibialis anterior and medial gastrocnemius in this study were compared to those obtained by other studies. Inoue et al. reported COI values during walking with and without a cane of 11 adult patients with CP [30]. The study by Inoue et al. differed from ours because it classified the gait cycle into two phases: the support phase and the swing phase. According to their study, the COI values of CP patients without a cane were 0.57 ± 0.09 in the stance phase and 0.46 ± 0.09 in the swing phase [30]. Our results show that the COI values on the operative side before HAL-SJ training were higher than those observed during the previous study (operative side COI in the stance phase: case 1, 0.77 ± 0.19, and case 2, 0.68 ± 0.21; operative side COI in the swing phase: case 1, 0.57 ± 0.21, and case 2, 0.54 ± 0.12). After training, COI values decreased to below the standards of adult CP patients during the stance phase in case 1 and during the stance and swing phases in case 2. Excessive co-contraction of the antagonist and agonist muscles increases joint stiffness and inhibits smooth movement. Therefore, it is important to maintain moderate co-contraction [23]. If HAL-SJ training facilitates functional recovery after equinus surgery, then it will shorten the rehabilitation period and reduce the economic burden experienced by the patient’s family. Additionally, if the risk of recurrence of equinus is reduced, then it would result in a significant economic benefit. However, whether the improvement in motor function will have an additional effect on the natural outcome of surgery remains unknown.

### Study Limitations

This manuscript is a case report, and the number of cases is not sufficient to prove the usefulness of HAL-SJ training after equinus corrective surgery. Furthermore, a comparative study with a control group is needed.

## 5. Conclusions

HAL-SJ training for pediatric patients after equinus corrective surgery resulted in motor function improvement. However, it is unknown whether the motor function improvement has further effects on the natural course during the postoperative period. Follow-up is necessary to observe the long-term outcomes of patients and determine whether HAL-SJ training has a preventive effect on equinus. Therefore, it is necessary to increase the number of cases and conduct comparative studies with control groups.

## Figures and Tables

**Table 1:** Clinical assessments.

			Case 1		Case 2	
			Pre	Post	Pre	Post
10-m walk test	Speed	m/s	0.59	1.05	0.71	0.82
	Stride length	m/s	0.19	0.38	0.32	0.33
	Cadence	steps/min	192.40	163.18	131.54	147.06
ROM	DKE	°	0	5	10	15
MAS	Dorsiflexion		0	0	0	0
	Plantar flexion		0	0	0	0
SCALE			10	12	12	12
GMFM	Standing	%	79.48	82.05	94.87	94.87
	Walking, running, and jumping	%	54.16	55.55	76.39	91.67

ROM: range of motion, DKE: dorsiflexion knee extension, MAS: Modified Ashworth scale, SCALE: selective control assessment of the lower extremity, GMFM: gross motor function measure, Post: after training, Pre: before training. ROM and MAS of the surgical side were assessed.

**Table 2:** Gait analysis.

Kinematic Parameters			Case 1		Case 2	
			Pre	Post	Pre	Post
Stance phase						
Maximum hip extension angle	°	Rt	−53.75	−17.71	−27.23	−34.72
		Lt	−42.03	−18.44	−41.23	−36.57
Maximum knee extension angle	°	Rt	−34.91	−27.61	−6.62	−18.62
		Lt	−29.99	−17.55	−12.72	−17.42
Swing phase						
Maximum hip flexion angle	°	Rt	66.47	59.04	77.78	76.16
		Lt	64.12	50.21	76.43	80.26
Maximum knee flexion angle	°	Rt	56.19	68.59	55.50	50.01
		Lt	65.83	69.62	53.16	50.71
Maximum ankle dorsiflexion angle	°	Rt	4.35	11.20	−4.26	9.90
		Lt	6.12	6.11	−8.50	1.82

The surgical side of case 1 is the right side. The surgical side of case 2 is the left side. Post: after training, Pre: before training.

**Table 3:** COI values.

		Case 1		Case 2	
		Pre	Post	Pre	Post
Ankle joint dorsiflexion	Rt	0.45 ± 0.31	0.88 ± 0.40	0.33 ± 0.15	0.57 ± 0.24
	Lt	0.58 ± 0.33	0.81 ± 0.23	Error	0.74 ± 0.27
Ankle joint plantar flexion	Rt	0.98 ± 0.58	0.26 ± 0.12	0.24 ± 0.21	0.21 ± 0.17
	Lt	0.38 ± 0.15	0.36 ± 0.16	Error	0.34 ± 0.27
COI value during walking					
Double stance 1	Rt	0.86 ± 0.32	0.91 ± 0.20	0.50 ± 0.17	0.54 ± 0.13
	Lt	0.89 ± 0.43	0.58 ± 0.18	0.78 ± 0.30	0.78 ± 0.21
Left stance phase/right swing phase	Rt	0.57 ± 0.21	0.65 ± 0.13	0.71 ± 0.20	0.48 ± 0.15
	Lt	0.57 ± 0.24	0.50 ± 0.06	0.68 ± 0.21	0.43 ± 0.06
Double stance 2	Rt	0.76 ± 0.25	0.63 ± 0.24	0.68 ± 0.16	0.59 ± 0.29
	Lt	0.53 ± 0.14	0.55 ± 0.09	0.63 ± 0.15	0.55 ± 0.11
Left swing phase/right stance phase	Rt	0.77 ± 0.19	0.48 ± 0.09	0.53 ± 0.23	0.43 ± 0.16
	Lt	0.50 ± 0.16	0.33 ± 0.07	0.54 ± 0.12	0.49 ± 0.09

The surgical side of case 1 is the right (Rt) side. The surgical side of case 2 is the left (Lt) side. COI: co-activation index, Post: after training, Pre: before training.
